# Reducing Work-Related Screen-Time in Healthcare Workers During Leisure Time (REDUCE SCREEN) – A Randomized Controlled Trial

**DOI:** 10.1007/s10916-026-02338-9

**Published:** 2026-01-17

**Authors:** Karsten Bartels, Karan Shah, Emelind Sanchez Rodriguez, Julie T. Hoffman, Megan L. Rolfzen, Juana Mora Valdovinos, Afton L. Hassett, Daniel I. Sessler

**Affiliations:** 1 Department of Learning Health Sciences, University of Michigan, Ann Arbor, MI USA; 2 Department of Psychiatry, University of Michigan, Ann Arbor, MI USA; 3 Department of Anesthesiology, University of Michigan, Ann Arbor, MI USA; 4 Department of Quantitative Health Sciences, Cleveland Clinic, Cleveland, OH USA; 5 Department of Anesthesiology, University of Nebraska Medical Center, Omaha, NE USA; 6 Center for Outcomes Research and Department of Anesthesiology, UTHealth Science Center, Houston, TX USA; 7 University of Michigan Medical School, 1540 E Medical Center Dr, Ann Arbor, MI 48109 USA

**Keywords:** Burnout, Stress, Perceived stress scale, Smartphone, Technology

## Abstract

The ubiquitous availability of work-related applications on personal devices makes healthcare workers prone to working during leisure time. We tested the hypothesis that an intervention to reduce work-related screen time during a weekend off reduces stress in healthcare workers in a pragmatic parallel design randomized controlled trial between November 2021 and November 2023. Healthcare workers using a smartphone with a work email application were eligible. Randomization was 1:1 to no treatment or a threefold educational intervention to: 1) activate automated responses to emails received, 2) reduce screen time, and 3) uninstall work applications from personal devices. The primary outcome was the change in participants’ stress from pre- to post-weekend, measured with the Perceived Stress Scale-10. The secondary outcome was device screen time. Among 815 enrolled participants, 520 responded to the post-intervention survey. The median [Q1, Q3] change from baseline Perceived Stress Scale-10 scores was -2 [-7, 0] in controls and -4 [-9, 0] in the intervention group. The mean difference (intervention – control) in post-intervention Perceived Stress Scale-10 scores, adjusted for baseline stress, was -1.6 (95% CI: -2.6, -0.6; *P* = 0.002). The median [Q1, Q3] change from baseline screen time was 0 [-2, 1] hours in the controls and -1 [-3, 0] hours in the intervention group. A three-pronged educational intervention targeting work-related screen time among healthcare workers doubled stress reduction during a non-work weekend. Stress reduction in the intervention group was mediated by reduced screen time. Future research should investigate long-term effects and broader implementation of such interventions to promote well-being in the healthcare workforce. Trial Registration: https://clinicaltrials.gov/study/NCT05106647. Identifier: NCT05106647, Registration date: November 4, 2021.

**Supplementary Information:**

The online version contains supplementary material available at 10.1007/s10916-026-02338-9.

## Introduction

Work-related stress can cause emotional exhaustion, a component of burnout that exacerbates healthcare worker attrition [1]. Indeed, in a survey study conducted in 2021 in 15,738 nurses and 5,312 physicians from 60 United States hospitals, high burnout was reported by 32% of physicians and 47% of nurses [2]. Stress-induced burnout increases the cost of care, promotes medical errors, lowers the quality of care, and reduces patient satisfaction [3]. Unsurprisingly, in 2021, the US healthcare workforce experienced an unprecedented 38% increase in healthcare workers exiting employment to non-healthcare sectors [4]. The burden of burnout has persisted globally since the COVID-19 pandemic accelerated social isolation and occupational disruption, further accelerating clinician labor shortages [5]. In response, leaders in the field have made concerted efforts to address burnout by reimagining health information technology, focusing on leveraging technology to increase job satisfaction and reduce stress [6, 7].

Electronic health record users report dissatisfaction with the increasing amount of time required for clerical tasks, greater cognitive burden, decreased physician work satisfaction, and reported high rates of professional burnout [8–10]. The omnipresence of handheld devices removes historical barriers that prevent engaging in work-related tasks during non-work periods. Indeed, in a 2021 cross-sectional study, 70% of clinicians did at least some work on a typical vacation day [11]. Outside of healthcare, employers have embraced approaches to protect leisure time for more than a decade. For example, to eliminate the issue of an overflowing inbox after time off, the German car manufacturer Daimler instituted a “Mail on Holiday” program that enabled employees to have all their incoming emails automatically deleted while they were on vacation [12].

There is a strong association between the amount of time healthcare workers spend on their screens to accomplish work-related tasks and subsequent stress and burnout [13]. Yet, it remains unknown if interventions aimed at reducing work-related screen time during leisure time reduce stress in healthcare workers. We, therefore, tested the hypothesis that a pragmatic three-pronged educational intervention aimed at reducing work-related screen time during a weekend off work reduces stress in healthcare workers. Secondarily, we tested the hypothesis that the educational intervention reduces leisure screen time on handheld devices.

## Methods

We conducted a parallel-design randomized trial to evaluate an educational intervention aimed at reducing work-related screentime in healthcare workers. The trial was reviewed by the University of Nebraska Medical Center Institutional Review Board (Protocol # 514–21-EX) and classified as exempt (educational, behavioral, and social science research under 45 CFR 46:104(d), category 2). Participants provided electronic informed consent before enrollment. All procedures followed the ethical standards of the responsible committee and with the Helsinki Declaration. Research Electronic Data Capture (REDCap) [14] was used to communicate with participants and track data. The trial was registered at ClinicalTrials.gov (NCT05106647) on November 4, 2021, before the first participant was enrolled. This report follows Consolidated Standards of Reporting Trials (CONSORT) guidelines [15].

### Study Population

**Fig. 1:**
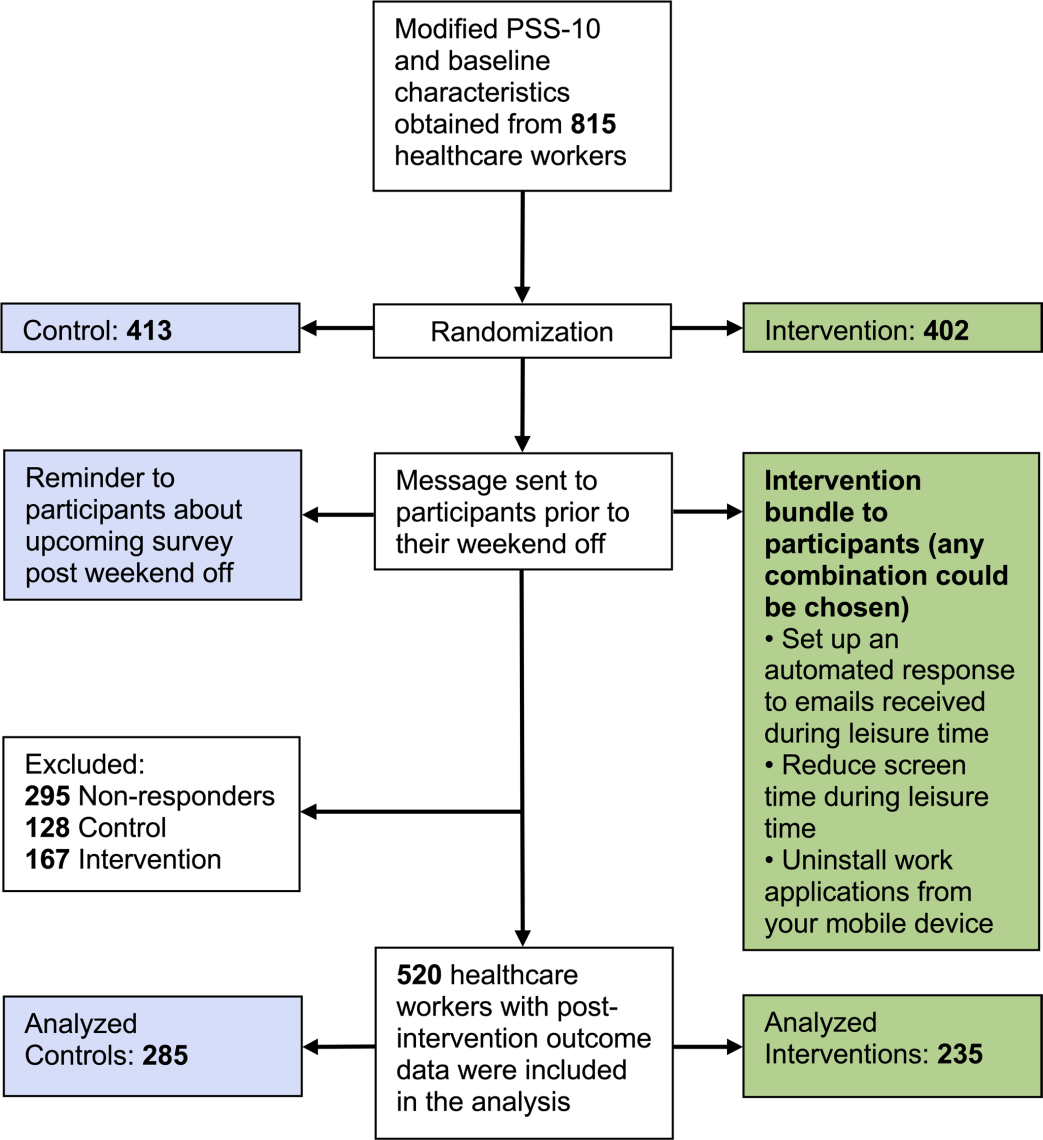
Study flow diagram

Adult healthcare workers who were able to read English, routinely used a smartphone, and had a work email application installed on their smartphone were eligible for enrollment. Coordinated from a United States academic anesthesiology department, participants were recruited using flyers, e.g., in the hospital cafeterias or at educational meetings, Quick Response (QR) codes embedded at grand rounds presentations, and a social media campaign (see Supplementary Information). From November 2021 to November 2023, we enrolled 815 healthcare workers (advanced practice providers, nurses, attending physicians, trainee physicians, and others), of whom 402 were randomized to our threefold educational intervention and 413 to no education. Among them, 295 did not respond to the post-intervention survey, so no outcome data could be collected. Our analysis set thus includes 520 workers, of whom 235 were assigned to the intervention, and 285 were assigned to the control (Fig. 1). The intake form included “other” categories for healthcare workers’ occupations other than advanced practice providers, nurses, and physicians (e.g., occupational, physical, and respiratory therapists, psychologists, or social workers). Similar to a hospital wellness program, ongoing participation was not mandated, and like most wellness programs, involvement was not compensated.

### Intervention

Healthcare workers were involved in the study's design by piloting the data collection tool and providing feedback on the choice of outcomes. Using the digital randomization function in the REDCap, participants were randomized 1:1 without stratification to our educational intervention or a reference group before a work-free weekend. The intervention group was provided the following threefold approach to reduce screen time during a selected weekend: 1) activate an automated response to emails received during leisure time, 2) reduce screen time during leisure time, and 3) uninstall work applications from personal mobile devices. The suite of interventions was presented through REDCap and virtual educational videos. Daily implementation of these interventions during the “off weekend” was strongly encouraged. Our education implementation strategy is informed by the COM-B model (Capability, Opportunity, Motivation-Behavior), which postulates that education interventions are most efficiently achieved through physical capability and reflective motivation [16]. Herein, we focused on increasing knowledge about the effects of screen time on well-being and offered the physical capability to have a work-free weekend. In the end, participants were free to follow all, some, or none of the threefold interventional strategies.

### Outcomes

Our primary outcome was participants’ post-intervention stress, adjusted for baseline stress. Stress was measured by the Perceived Stress Scale (PSS)−10, a 10-item self-report measure that is widely used to assess the degree to which life situations are stressful [17]. PSS-10 scores are consistent over time with repeated testing and are a reliable measure of psychological stress over various ages, education levels, and incomes [18]. The score ranges from 0–40, with higher values indicating worse stress [18].

The PSS-10 was administered to participants twice using REDCap infrastructure, once at enrollment and a second time on Monday after their trial weekend off. In case of non-response to the Monday message, participants received a daily reminder message for up to three additional subsequent days.

Our secondary outcome was average daily personal device screen time use during the trial weekend, adjusted for baseline screen time exposure. Based on feedback in preliminary testing, personal device daily screen use was not recorded in minutes but recorded from the reporting functions built into participants’ handheld devices and was reported on an hourly 11-point ordinal scale ranging from “0 to < 1 h” to “ > 10 h”.

### Statistical Analysis

Analysts were masked to group allocation until the primary analysis was complete. Given the nature of the study, participants could not be masked, but they were not informed whether we considered their assigned exposure intervention or lack thereof to be treatment versus control.

Analyses were performed using a modified intention-to-treat approach, with the modification being that participants with missing outcome data had to be excluded. The primary analysis, the effect of the intervention on stress, was performed by fitting a linear regression model with the assigned intervention group as the primary covariate and the post-intervention PSS-10 score as the outcome, adjusted for baseline PSS-10 scores. For the secondary analysis, the effect of the intervention on self-reported screen time was assessed by fitting an ordinal regression model with the reversed average post-intervention screen time as the outcome (so that a higher value corresponds to a lower average screen time) and the assigned intervention group as the primary covariate of interest, adjusted for reported baseline screen time. Exploratory analyses included the association between self-reported screen time and stress, which was assessed using a linear regression model, and the effect of the intervention based on which of the three interventions (activate an automated response to emails received during leisure time, reduce screen time during leisure time, and uninstall work applications from personal mobile devices) were adhered to. In line with the pragmatic design, participants randomized to the intervention group could choose to follow any combination or none of the suggested interventions, similar to other hospital wellness programs [19]. Given that the choice of which of the suggested screen-time limiting approaches to follow was left to the discretion of the participant in the intervention group, the exploratory analyses are observational in nature. Statistical significance was set at a level of *P* < 0.05. No multiple testing correction was applied, and thus, the non-primary analyses should be interpreted as exploratory in nature. All analyses were conducted using R statistical software (version 4.3.1; R Core Team 2023).

Based on a similar study, we assumed a baseline mean PSS-10 score of 20 with a standard deviation of 7 [20]. We defined a reduction in stress of 10% from baseline as clinically meaningful after the trial weekend, estimating a mean score of 18 and a standard deviation of 7 for the intervention group [21]. We estimated that 194 healthcare workers per group would provide 80% power for detecting a significant difference in PSS-10 score at an alpha of 0.05 using G-Power 3.1 [22].

## Results

Of the 815 enrolled participants (intervention: 402, control: 413), 520 (63.8%) completed the post-intervention survey. Despite participant attrition, the final sample size was greater than the initially anticipated sample size of 194 per group and thus provided sufficient power for detecting clinically meaningful differences in PSS-10 scores. Baseline characteristics, including PSS-10 scores, in participants who completed the study (Table 1) versus those who did not (Supplemental Table 1) were not significantly different between groups. Of the respondents who completed the post-intervention survey, three subjects had missing information about the secondary outcome (average screen time). They were included in the primary analysis but excluded from the secondary analysis. More women (57%) than men participated. The modal age group was 25–34 years old, and the modal worker occupation was nursing (Table 1). Of the 520 participants, 62 (11.9%) were Advanced Practice Providers, 178 (34.2%) nurses, 86 (16.5%) attending physicians, and 56 (10.7%) trainee physicians.

**Table 1:** Participant baseline characteristics

Characteristic	Control	Intervention
*n*	285	235
Age, *n* (%)		
* 19–24*	44 (15.4)	30 (12.8)
* 25–34*	123 (43.2)	107 (45.5)
* 35–44*	75 (26.3)	59 (25.1)
* 45–54*	31 (10.9)	25 (10.6)
* 55–64*	7 (2.5)	12 (5.1)
* 65 and over*	4 (1.4)	2 (0.9)
* Declined to answer*	1 (0.4)	0 (0.0)
Gender, *n* (%)		
* Men*	116 (40.7)	106 (45.1)
* Woman*	167 (58.6)	128 (54.5)
* Other*	2 (0.7)	1 (0.4)
* Declined to answer*	0 (0.0)	0 (0.0)
Type of healthcare worker, *n* (%)		
* Advanced Practice Provider*	30 (10.5)	32 (13.6)
* Nurse*	107 (37.5)	71 (30.2)
* Physician (attending)*	49 (17.2)	37 (15.7)
* Physician (resident or fellow)*	30 (10.5)	26 (11.1)
* Other*	69 (24.2)	68 (28.9)
* Declined to answer*	0 (0.0)	1 (0.4)
Baseline PSS-10, median [Q1, Q3]	17 [11, 22]	18 [13, 22]
Baseline average screen time, median [Q1, Q3]	6 [5, 8]	6 [4, 8]

PSS-10, Perceived Stress Scale 10. “Other” for gender included self-identified non-binary genders other than men or women. “Other” for healthcare workers’ occupations other than advanced practice providers, physicians, and nurses. Screen time was reported on an hourly 11-point ordinal scale

**Fig. 2:**
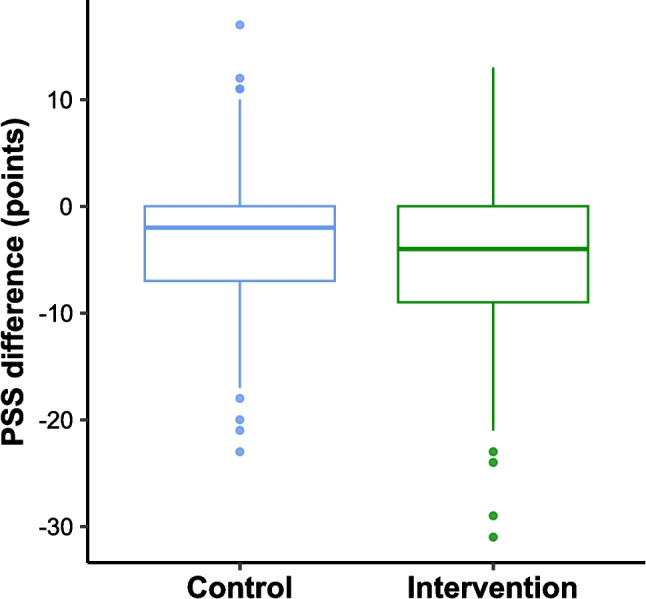
Difference in post-intervention and baseline PSS-10 scores. The central line in the box corresponds to the median, and the lower and upper hinges correspond to the first and third quartiles. The lower whisker is no more than 1.5 times the interquartile range (IQR) from the lower hinge, and the upper whisker is no more than 1.5 times the IQR from the upper hinge. The points correspond to data lying beyond the lower or upper whiskers. Negative scores indicate a reduction in stress from baseline

The median [Q1, Q3] baseline PSS-10 scores were 17 [11, 22] points in the control group and 18 [13, 22] points in the intervention group. The median [Q1, Q3] change from baseline PSS-10 scores (*n* = 520) was −2 [−7, 0] in the control group and −4 [−9, 0] in the intervention group (Fig. 2). The mean difference in baseline-adjusted post-weekend PSS-10 scores, estimated using a linear regression model, was −1.6 (95% CI: −2.6, −0.6; *P* = 0.002).

**Fig. 3:**
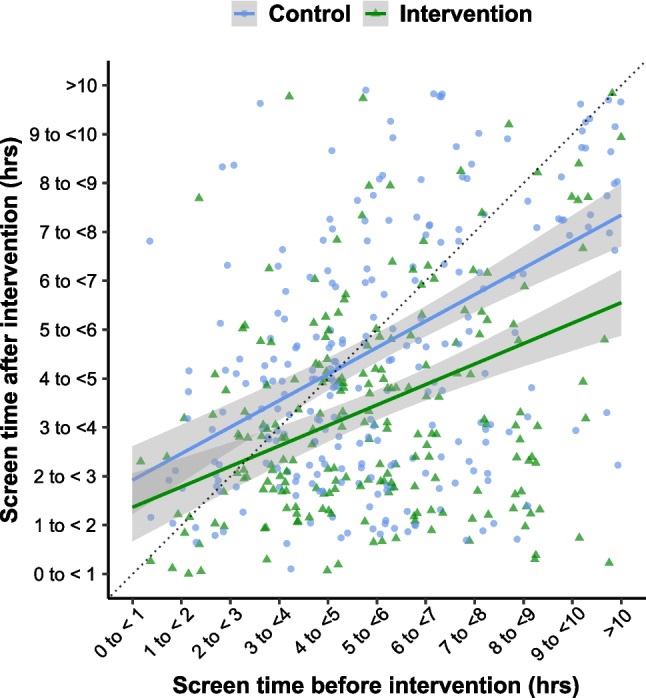
Average screen time before and after intervention. The dotted line represents no effect. Points above the dotted line indicate an increase in screen time from baseline, while points below the line indicate a reduction in average screen time. Given the ordinal nature of the data, random noise was added to the data points to avoid overplotting

Average screen time (work- and leisure-related) during the off weekend was measured on an ordinal scale using hourly intervals (0- < 1 h, 1- < 2 h, up to > 10 h). The median [Q1, Q3] average screen time at baseline was 5- < 6 [4- < 5, 7- < 8] hours in the control group and 5- < 6 [3- < 4, 7- < 8] hours in the intervention group. The median [Q1, Q3] change from baseline average screen time was 0 [−2, 1] in the control group and −1 [−3, 0] in the intervention group (Fig. 3). The ordinal regression model (*n* = 517) estimated an odds ratio (intervention/control) for a decrease in screen time of 2.29 (95% CI: 1.68, 3.12; *P* < 0.001). In other words, participants randomized to the intervention group had 129% increased odds of having lower average screen time post-intervention.

An exploratory analysis showed that among workers in the interventional group, stress was most reduced in workers who uninstalled work applications (*n* = 84), with a mean (95% CI) difference of −3.1 (−5.0, −1.2) points compared to those who did not follow any interventions (*n* = 16). (Table 2) The mean (95% CI) difference for those who reduced screen time (*n* = 186) was −1.0 (−3.2, 1.1) points, and for those who set up automatic out-of-office email replies (*n* = 104), it was 1.1 (−0.7, 2.9) points compared to those who did not follow any interventions (*n* = 16).

**Table 2:** Participant intervention choices

	Email autoreply	Reduce screen time	Uninstall apps
Email autoreply	13	38	3
Reduce screen time	38	84	14
Uninstall apps	3	14	17
All 3 recommendations: 50 subjects; None of the recommendations: 16 subjects			

Summary of which of the suggested recommendations to reduce screen time were chosen among participants randomized to the three-pronged educational intervention. Participants were free to follow all, some, or none of the threefold interventional strategies

## Discussion

Leisure activities improve health via psychological, biological, social, and behavioral mechanisms [23]. It is, therefore, unsurprising that a leisure weekend in and of itself reduced stress in healthcare workers. Specifically, the median reduction from baseline PSS-10 scores was two points in untreated reference workers, corresponding to a 12% stress reduction from baseline after a weekend off. In contrast, workers assigned to our educational program had a 4-point reduction, corresponding to a 23% decrease in stress scores. Education regarding theory-driven, behavior change interventions to reduce screen time thus almost doubled the stress relief accrued over a non-work weekend.

Strengths of the study include its pragmatic design, large sample size, and the use of a well-validated primary outcome measure, the PSS-10 [17]. Although we could not determine what fraction of residual screen time was work-related versus leisure activity, the overall reduced stress was presumably mediated by decreased screen time consequent to the education offered, given that it was the only difference between groups. Total screen time was reduced in the intervention group by about an hour. The relationship between screen time and observed reductions in stress levels is likely non-linear and dependent on the type of screen activities. However, outside of medicine, it is documented that screen time in adolescents is associated with perceived stress (β = 0.27; 95% CI, 0.13 to 0.41) and depressive symptoms (β = 0.30; 95% CI, 0.15 to 0.44) [24], alluding to the benefits of generalized screen time reduction, work-related or otherwise. Although we targeted the healthcare environment for this study, the described approach and intervention are easily scalable to community-based contexts.

As might be expected, we observed the most substantial stress reductions in participants who uninstalled work applications, thus completely disconnecting from work activities. Uninstalling work-related applications might also have been perceived as providing greater autonomy. This could improve an unfavorable ratio of high workload demands with low decision latitude, a known driver of burnout [25]. Uninstalling work applications was the most drastic of our suggested interventions, and it seems likely that participants who chose to do so were more motivated than others to disengage from their screens. Regardless of potential mechanisms of the individual components of our educational intervention, simply being asked to reduce screen time provided about the same benefit on PSS-10 scores as practicing yoga does for people with chronic back pain [21]. While this work provides evidence of preliminary effectiveness, further implementation strategies, such as the development of persistent academic partnerships and a formal blueprint [26], are needed to inform a sustainable tiered strategy of system-wide adoption that minimizes the burden of (de)installation, while efficiently managing missed messages and emergent patient care.

In this study, we intentionally offered a diverse sample of healthcare workers the option to select among three different interventions (setting up automated out-of-office email replies, uninstalling work-related applications, and/or reducing overall time spent on the screen). This approach enabled participants to choose whichever approach would work best for their individual circumstances on their selected weekend off clinical duty. Guided by the COM-B framework for understanding behavior change [16], we identified physical capability and reflective motivation as key components to optimize the impact of educational interventions. Accordingly, we aimed to enhance participants’ capability by increasing their knowledge regarding screen time and its negative effects, providing opportunities for behavior change by offering practical strategies and environmental cues to reduce work-related screen use, and motivating via messaging designed to cultivate intrinsic desire for change (i.e., improved well-being). Given the high likelihood of future healthcare crises, the personalization of interventions aimed at mitigating stress and burnout and healthcare is pertinent. This approach aligns with the call to expand the Triple Aim for health system performance - enhancing patient experience, improving population health, and reducing costs - to the Quadruple Aim, which incorporates the critical objective of improving the work-life of healthcare providers [27].

## Limitations

The primary limitation is that a third of randomized participants did not complete the post-weekend off assessment, and, therefore, could not be included in our primary outcome analysis. While it is possible that participants who did not follow any of the suggestions were less likely to complete our survey, baseline characteristics, including baseline PSS-10 scores, were similar in participants who completed the study and those who did not. However, it is important to recognize that the potential introduction of attrition bias may still impact the external validity. At study inception, we considered offering an incentive to complete the study but decided against it. While we deliberately aimed to include intrinsically motivated participants [28], we also aimed to conduct a pragmatic trial that would mimic conditions existing in a health system implementing screentime-reducing measures in their workers [29, 30]. For example, uninstalling an electronic health record app before leisure time may be undesirable for clinicians and may have unintended consequences related to missed messages or emergent patient care. Yet, a health system may be able to adjust incentive programs for timely chart closure to account for scheduled time off as part of operational programs designed to reduce burnout in clinicians [31]. Furthermore, while our primary outcome was a validated measure of perceived stress, our secondary outcome relied on self-reported data, which was based on mobile device operating system-generated metrics. Future iterations could consider mobile device management profile installations, third-party apps, or remote digital trace logging to collect more objective and granular measures of screen-time data [32].

## Conclusion

A three-pronged educational intervention aimed at reducing work-related screen use during scheduled leisure time doubled the reduction of stress levels in healthcare workers following a weekend off. The decrease in stress levels from pre-weekend perceived stress to post-weekend perceived stress was mediated by a reduction in screen time and was most prominent in healthcare workers who chose to remove work-related applications from their personal communication devices.

## Supplementary Information

Below is the link to the electronic supplementary material.

Supplementary file1 (DOCX 4090 KB)

## Data Availability

Deidentified data will be available at Dryad (10.5061/dryad.ghx3ffbz4) within 30 days of publication.

## Declarations

### Access to Data

Deidentified data will be made publicly available at Dryad (10.5061/dryad.ghx3ffbz4) within 30 days of publication.
