# Supplementary material for: Reducing Work-Related Screen-Time in Healthcare Workers During Leisure Time (REDUCE SCREEN) – A Randomized Controlled Trial
Source: J Med Syst. 2026 Jan 17;50(1):11. doi: 10.1007/s10916-026-02338-9 (PMC12811315; doi:10.1007/s10916-026-02338-9)
Supplement: Supplementary file 1 — Supplementary file1 (DOCX 4090 KB) [file 10916_2026_2338_MOESM1_ESM.docx]

Supplementary Information for “**Reducing work-related screen-time in healthcare workers during leisure time (REDUCE SCREEN) – a randomized controlled trial”**

| **Supplement Item** | Page |
| --- | --- |
| **Supplemental Graphical Abstract** | 2 |
| **Supplemental Table 1. Non-responder baseline characteristics.** | 3 |
| **Supplemental Methods 1. Healthcare worker recruitment.** | 4 |
| **Supplemental Methods 2. Enrollment survey iterations.** | 5 |
| **Supplemental Methods 3. Intervention material.** | 8 |
| **Supplemental Methods 4. Secondary outcome report.** | 9 |
| **Supplemental Data. Physician specialties.** | 10 |

#
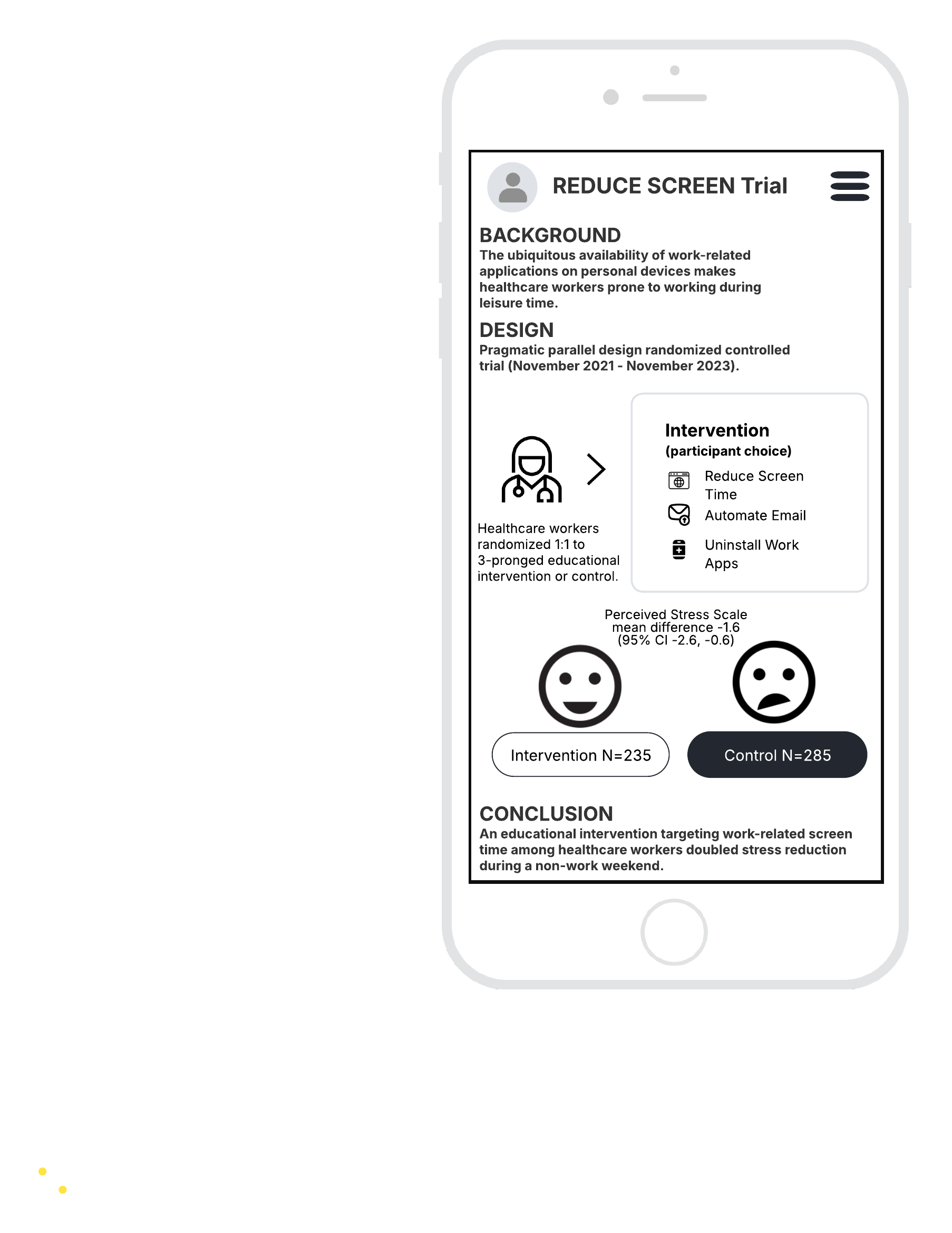
Supplemental Graphical Abstract

## Supplemental Table 1. Non-responder baseline characteristics.

| **Characteristic** | **Control** | **Intervention** |
| --- | --- | --- |
| Participants | 128 | 167 |
| Age, n (%) |  |  |
| *19-24* | 18 (14.1) | 22 (13.2) |
| *25-34* | 48 (37.5) | 70 (41.9) |
| *35-44* | 37 (28.9) | 50 (29.9) |
| *45-54* | 14 (10.9) | 15 (9.0) |
| *55-64* | 10 (7.8) | 10 (6.0) |
| *>65* | 1 (0.8) | 0 (0.0) |
| *Declined to answer* | 0 (0.0) | 0 (0.0) |
| Gender, n (%) |  |  |
| *Male* | 43 (33.6) | 48 (28.7) |
| *Female* | 85 (66.4) | 119 (71.3) |
| *Other* | 0 (0.0) | 0 (0.0) |
| *Declined to answer* | 0 (0.0) | 0 (0.0) |
| Type of healthcare worker, n (%) |  |  |
| *Physician (attending)* | 20 (15.6) | 20 (12.0) |
| *Physician (resident or fellow)* | 16 (12.5) | 15 (9.0) |
| *Advanced Practice Provider* | 12 (9.4) | 26 (15.6) |
| *Nurse* | 39 (30.5) | 56 (33.5) |
| *Other* | 41 (32.0) | 49 (29.3) |
| *Declined to answer* | 0 (0.0) | 1 (0.6) |
| Baseline average screen time, median [Q1, Q3] | 6 [5, 8] | 5 [4, 7] |
| Baseline PSS-10, median [Q1, Q3] | 18 [13, 21] | 19 [14, 23] |

PSS-10, Perceived Stress Scale 10. “Other” for gender included self-identified non-binary genders other than men or women. “Other” for healthcare workers’ occupations other than advanced practice providers, physicians, and nurses. Baseline average screen time was measured on an ordinal scale using hourly intervals.

## Supplemental Methods 1. Healthcare worker recruitment template example posted on flyers and QR codes in hospital-facing environments and social media platforms such as Facebook and Twitter/X.


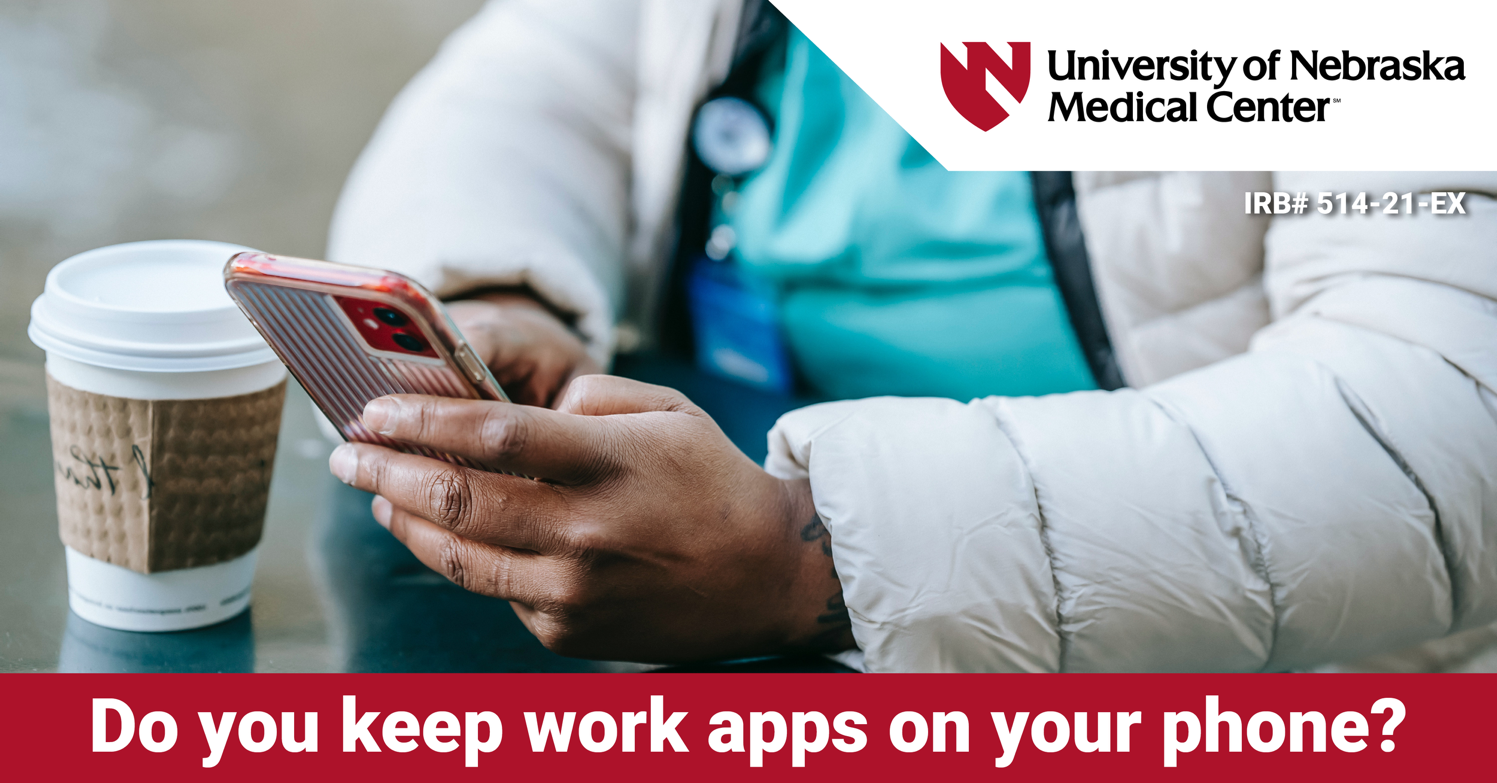


## Supplemental Methods 2. Enrollment survey iterations.

**STUDY SUBJECT ELIGIBILITY CHECKLIST**

| **Protocol Number and Study Title** | # 514-21-EX. REDUCE SCREEN: Reducing work-related screen-time in healthcare workers during leisure time (REDUCE SCREEN) – a randomized controlled trial |
| --- | --- |
| **Protocol Version & Date** | Protocol Version 1.0 2021JUN |
| **Baseline Survey Collection** | Age, gender, inclusion criteria below, weekend off determination, baseline PSS-10 |

Participants must meet all of the following inclusion and exclusion criteria to be eligible for participation in this study.

Refer to protocol to verify baseline test.

# Inclusion Criteria

(**Reminder:** All answers must be answered Yes or N/A for the subject to be eligible.)

| **Yes** | **No** | **N/A** | **Inclusion Criteria** | **Criteria**  **Answer** | **Date Criteria Completed** |
| --- | --- | --- | --- | --- | --- |
|  |  |  | 1. Active healthcare worker. |  |  |
|  |  |  | 2. Greater than or equal to 19 years of age. |  |  |
|  |  |  | 3. Routinely use a smartphone; have access to smartphone. |  |  |
|  |  |  | 4. Able to read English. |  |  |
|  |  |  | 5. Have work application installed on smartphone (e.g., Outlook, email, Haiku, etc.) |  |  |

# Informed Consent

This study seeks to understand the effect that work-related screentime has on health care worker stress. As

communication technology is increasingly used in health care, the line between work and leisure time is blurred. Health care workers are often obliged to communicate with patients and peers outside of working hours through email applications, consequently increasing work-related screen time. We seek to understand if reducing work-related screen time reduces stress levels in health care workers.

Completing the survey is equivalent to you giving consent. No personally identifiable information will be collected and data will only be available to personnel conducting the study. Thank you for your participation.

**STUDY SUBJECT ELIGIBILITY CHECKLIST**

| **Protocol Number and**  **Study Title** | # 514-21-EX. REDUCE SCREEN: Reducing work-related screen-time in healthcare workers during leisure time (REDUCE SCREEN) – a randomized controlled trial |
| --- | --- |
| **Protocol Version & Date** | Protocol Version 2.0 2021JUL |
| **Baseline Survey Collection** | Age, gender, inclusion criteria below, type of healthcare provider, specialty designation, baseline average daily screen time, weekend off determination, baseline PSS-10 |

Participants must meet all of the following inclusion and exclusion criteria to be eligible for participation in this study. Refer to protocol to verify baseline test.

# Inclusion Criteria

(**Reminder:** All answers must be answered Yes or N/A for the subject to be eligible.)

| **Yes** | **No** | **N/A** | **Inclusion Criteria** | **Criteria**  **Answer** | **Date Criteria Completed** |
| --- | --- | --- | --- | --- | --- |
|  |  |  | 1. Active healthcare worker. |  |  |
|  |  |  | 2. Greater than or equal to 19 years of age. |  |  |
|  |  |  | 3. Routinely use a smartphone; have access to smartphone. |  |  |
|  |  |  | 4. Able to read English. |  |  |
|  |  |  | 5. Have work application installed on smartphone (e.g., Outlook, email, Haiku, etc.) |  |  |

# Informed Consent

This study seeks to understand the effect that work-related screentime has on health care worker stress. As

communication technology is increasingly used in health care, the line between work and leisure time is blurred. Health care workers are often obliged to communicate with patients and peers outside of working hours through email applications, consequently increasing work-related screen time. We seek to understand if reducing work-related screen time reduces stress levels in health care workers.

Completing the survey is equivalent to you giving consent. No personally identifiable information will be collected and data will only be available to personnel conducting the study. Thank you for your participation.

**STUDY SUBJECT ELIGIBILITY CHECKLIST**

| **Protocol Number and**  **Study Title** | # 514-21-EX. REDUCE SCREEN: Reducing work-related screen-time in healthcare workers during leisure time (REDUCE SCREEN) – a randomized controlled trial |
| --- | --- |
| **Protocol Version & Date** | Protocol Version 3.0 2021AUG |
| **Baseline Survey Collection** | Age, gender, inclusion criteria below, type of healthcare provider, specialty designation, baseline average daily screen time (ordinal hourly intervals), weekend off determination, baseline PSS-10 (reworded timing from month to 2 days) |

Participants must meet all of the following inclusion and exclusion criteria to be eligible for participation in this study. Refer to protocol to verify baseline test.

# Inclusion Criteria

(**Reminder:** All answers must be answered Yes or N/A for the subject to be eligible.)

| **Yes** | **No** | **N/A** | **Inclusion Criteria** | **Criteria**  **Answer** | **Date Criteria Completed** |
| --- | --- | --- | --- | --- | --- |
|  |  |  | 1. Active healthcare worker. |  |  |
|  |  |  | 2. Greater than or equal to 19 years of age. |  |  |
|  |  |  | 3. Routinely use a smartphone; have access to smartphone. |  |  |
|  |  |  | 4. Able to read English. |  |  |
|  |  |  | 5. Have work application installed on smartphone. (e.g., Outlook, email, Haiku, etc.) |  |  |

# Informed Consent

This study seeks to understand the effect that work-related screentime has on health care worker stress. As

communication technology is increasingly used in health care, the line between work and leisure time is blurred. Health care workers are often obliged to communicate with patients and peers outside of working hours through email applications, consequently increasing work-related screen time. We seek to understand if reducing work-related screen time reduces stress levels in health care workers.

Completing the survey is equivalent to you giving consent. No personally identifiable information will be collected and data will only be available to personnel conducting the study. Thank you for your participation.

## Supplemental Methods 3. Intervention material.

**Educational Videos:**

1. [TEDx](https://www.youtube.com/watch?v=m1_QlV6XCNs&t=28s) | Learning to look up again – controlling your smartphone addiction | Ross Sleight

**
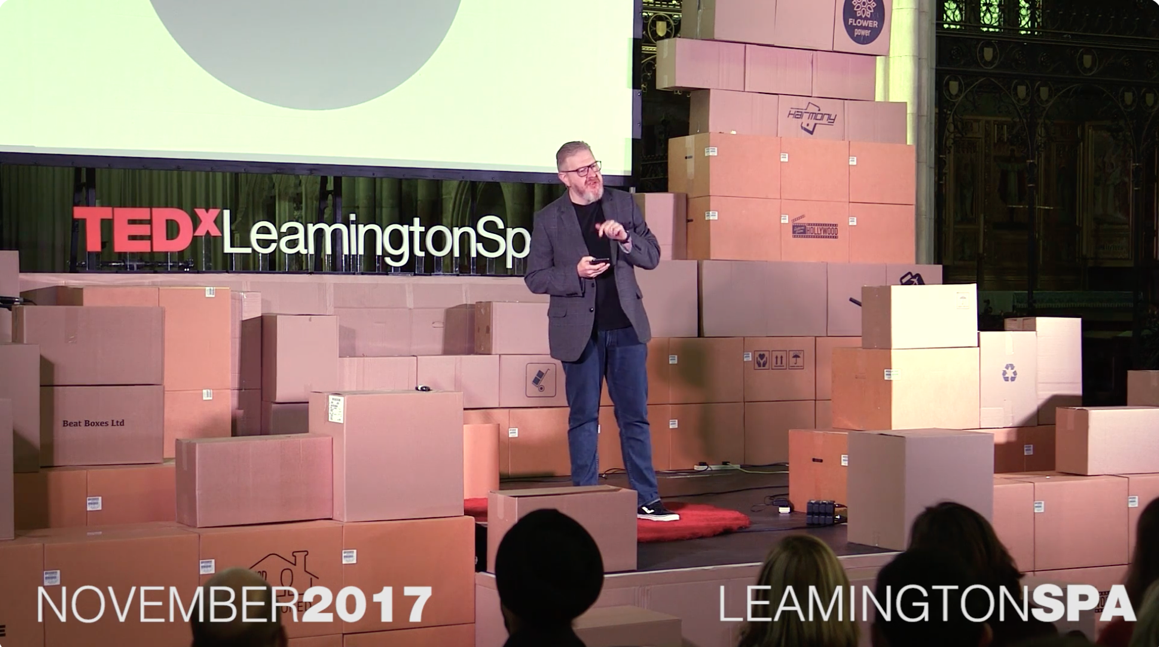
**

1. [Insider Science](https://www.youtube.com/watch?v=_1V0rDSTC9I&t=1s)| How Smartphones Affect Your Sleep | Dr. Dan Siegel


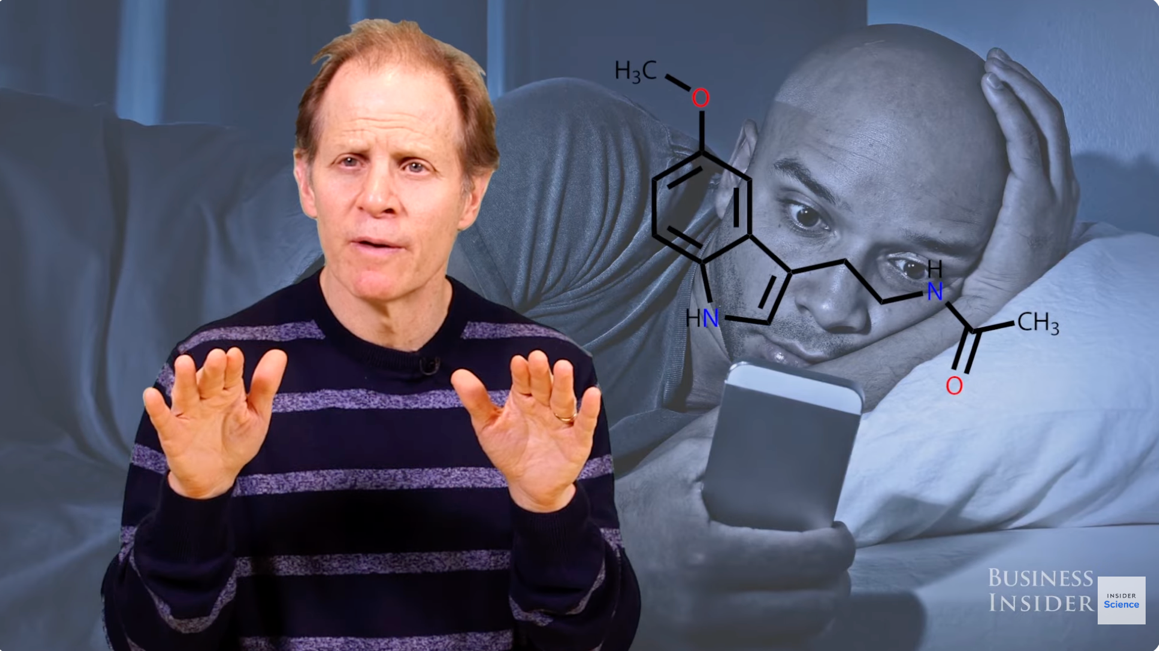


**Suggested Automated Email Prompt:**


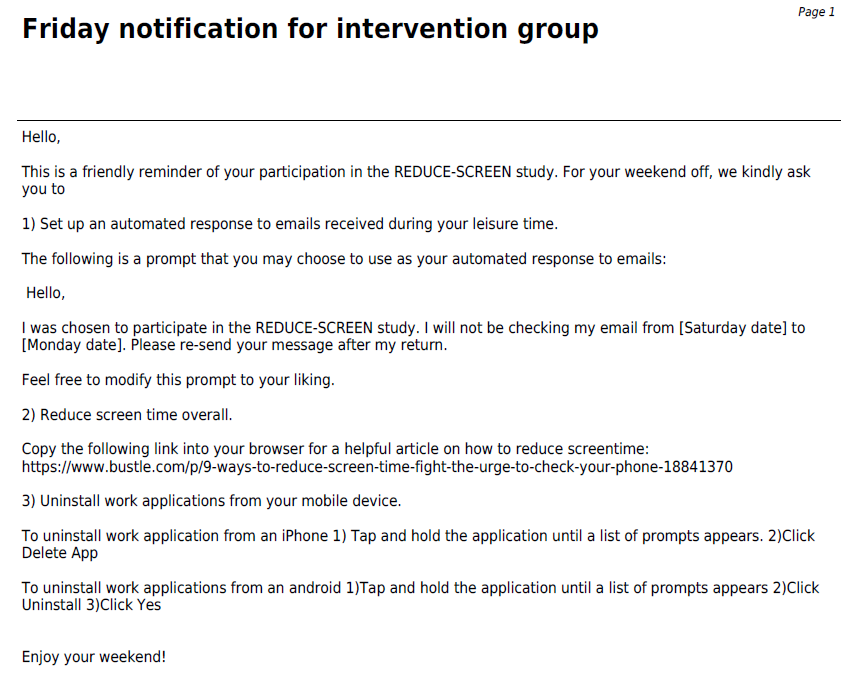


Supplemental Methods 4. Secondary outcome report instruction for iOS and Android operating systems. Self-report data were subsequently entered and securely stored in REDCap.


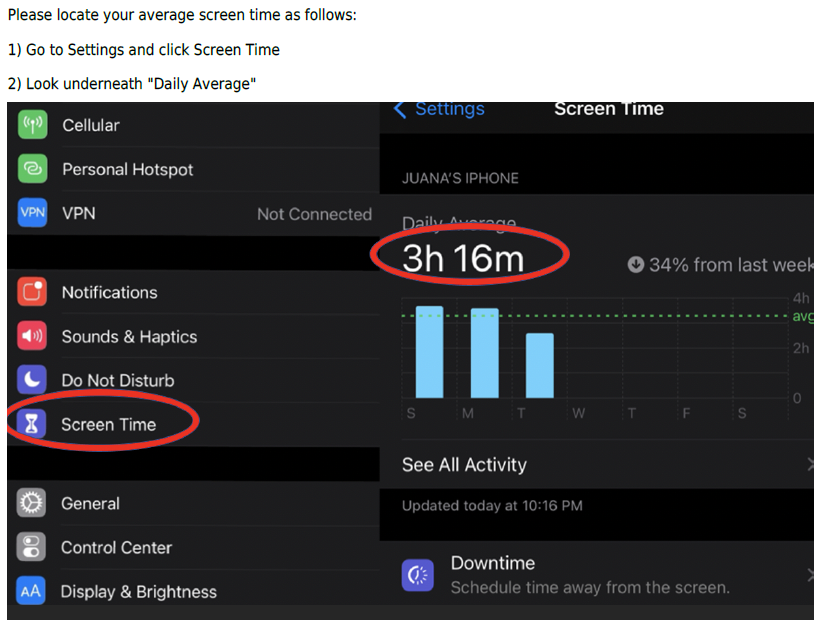


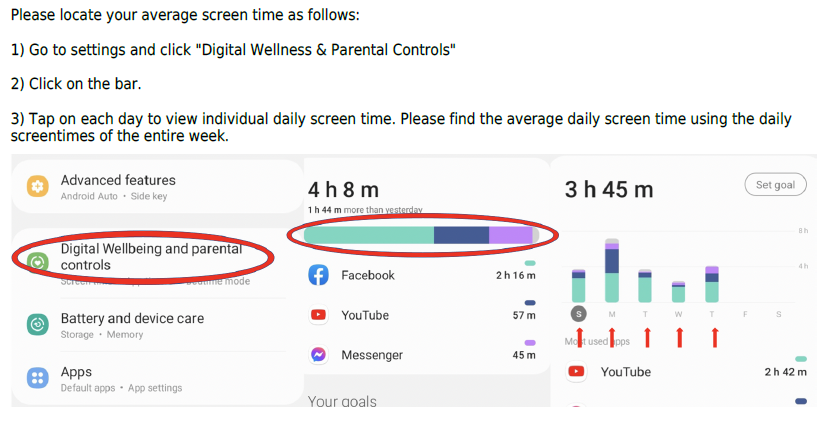


# Supplemental Data. Physician specialties.

| **Specialty** | **Control** | **Intervention** |
| --- | --- | --- |
| Family Medicine | 20 | 12 |
| Internal Medicine | 11 | 14 |
| Pediatrics | 10 | 14 |
| Anesthesiology | 7 | 12 |
| Emergency Medicine | 6 | 2 |
| Psychiatry | 6 | 3 |
| Surgery | 6 | 1 |
| Obstetrics & Gynecology | 4 | 2 |
| Radiology | 4 | 0 |
| Neurology | 2 | 2 |
| Other | 2 | 1 |
| Missing | 1 | 0 |
